# Unilateral Horner’s Syndrome and Upper Limb Paresthesia Following Labor Epidural Analgesia: A Case Report

**DOI:** 10.7759/cureus.75931

**Published:** 2024-12-18

**Authors:** Amr Barghout, Ahmed Rashwan

**Affiliations:** 1 Anesthesia, Medway NHS Foundation Trust, Kent, GBR

**Keywords:** compli, horner’s syndrome, labour epid, local anesthetic, pregnan

## Abstract

Horner’s syndrome arises from a disruption in the sympathetic nervous system. Although it is an uncommon complication of labor epidural analgesia, its occurrence is significantly more frequent among pregnant women. The incidence of Horner's syndrome after epidural analgesia for labor is 0.4%. This heightened frequency may be attributed to the extensive upward spread of local anesthetic (LA), which is possibly amplified by the anatomical and physiological changes during pregnancy. Here, we describe a case of unilateral Horner’s syndrome following epidural analgesia, which was also associated with unilateral upper limb paresthesia.

## Introduction

Horner’s syndrome, also referred to as oculo-sympathetic palsy, occurs due to a disruption in the sympathetic nervous system. It is characterized by a triad of symptoms: ptosis, myosis, and anhydrosis on the affected side. Benign or malignant tumors of the nervous system or the adjacent structures, trauma, or cardiovascular diseases can cause this syndrome, or it can occur as a hereditary or iatrogenic disease [1].

Although labor epidural analgesia is an uncommon cause of Horner’s syndrome and typically, this condition is benign and self-limiting once the local anesthetic (LA) is metabolized in the body, its incidence is notably higher among pregnant women [2]. This increased incidence can be attributed to several anatomical and physiological changes during pregnancy, such as the narrowing of the epidural space due to venous engorgement and the temporary rise in epidural pressure from uterine contractions. Additionally, elevated progesterone levels during pregnancy may sensitize neuronal tissue, enhancing the blockade of the sympathetic nervous system [3]. 

This case report aims to present an instance of recurrent Horner's syndrome accompanied by upper limb paresthesia and motor weakness, a rare complication following labor epidural analgesia.

## Case presentation

A 26-year-old pregnant woman with a body mass index (BMI) of 21.6 kg/m², a height of 174 cm, and a weight of 65.4 kg, presented to the labor ward at 39 weeks and 5 days' gestation with regular contractions. She was a primigravida with no significant medical history or drug allergies. Upon initial examination, her cervix was dilated to 6 cm. Despite using a mixture of 50% nitrous oxide and 50% oxygen, she remained uncomfortable and requested epidural labor analgesia.

The epidural procedure was performed two hours later due to the on-call anesthetist being occupied in the operating theater. Using a 16G Tuohy needle, the epidural space was located at the L4-L5 interspace in a sitting position with the loss of resistance to saline at 6 cm from the skin. An epidural catheter was threaded and fixed at a depth of 11 cm from the skin, with 5 cm in the epidural space. A positive meniscus drop test confirmed the catheter’s appropriate positioning and negative aspiration was confirmed before injecting a test dose of 3 mL of 0.1% levobupivacaine and 2 mcg/mL fentanyl. The patient remained vitally stable, and a loading dose of 12 mL of the same mixture was administered. She was then positioned in Fowler’s position, and patient-controlled epidural analgesia (PCEA) was set up according to hospital policy with a mixture of 0.1% levobupivacaine and 2 mcg/mL fentanyl, allowing 10 mL boluses with a 20-minute lockout time and no continuous infusion background.

Within 20 minutes, the patient reported feeling more comfortable, with contractions becoming less painful. She experienced no motor block or other neurological symptoms initially and used the PCEA to the maximum allowed boluses per hour. However, after the first hour, her partner noticed ptosis (*lazy eye*) on her right side, which was reported to the midwife and escalated to the on-call anesthetist. Upon review, the block height was at T10 with good pain control, a Bromage score of 0, equal eyelid movements, and equal pupil sizes. An hour later, the patient complained of increasing numbness in her right breast and arm. Further examination revealed a block height of T1 on the right side and T8 on the left side, with unilateral miosis, right upper eyelid ptosis, and decreased handgrip strength (4/5) on the right side. A sensory block was noted on the medial aspect of the arm at the T1 dermatome. Her maternal arterial pressure was 124/63 mmHg, and cardiotocography showed accepted fetal heart rate and variability.

The symptoms suggested a cephalad spread of the LA affecting the superior cervical sympathetic chain and the brachial plexus. The patient was reassured and monitored closely. The PCEA settings were adjusted, reducing the bolus dose from 10 to 7 mL. She remained hemodynamically stable, and her neurological symptoms gradually regressed over six hours. As labor progressed to 9 cm dilation, the patient opted for a cesarean section. The epidural catheter was removed, and a spinal block was performed at the same level using 2 mL of hyperbaric bupivacaine 0.5% and 500 mcg of diamorphine. The surgery was uneventful, and the patient was discharged to recovery in stable condition. Postoperatively, she had no residual sensory or motor block, and the symptoms of Horner’s syndrome resolved. She was advised that despite the rare complication, future epidural analgesia could still be considered. The patient continued her management in the ward according to the obstetrics plan and was discharged home the following day.

## Discussion

Horner’s syndrome occurs due to the disruption of sympathetic pre-ganglionic fibers, typically originating from C8 to T1 and occasionally extending as low as T4. We hypothesize that a rupture in the dura mater during the initial epidural procedure may have allowed for greater epidural spread during subsequent epidural boluses [4]. This complication likely arose due to the anatomical and physiological changes associated with pregnancy, such as increased epidural pressure [5] and venous engorgement.

The occurrence of ipsilateral upper arm paresthesia is difficult to explain but may be attributed to factors such as a midline septum narrowing the epidural space or the baricity of the LA. Also, the presence of a midline septum has been suggested to be a cause [6]. Typically, the symptoms of Horner’s syndrome are transient, with an average onset of 25 minutes and a mean duration of 215 minutes. In this case, symptoms manifested 50 minutes after the initial bolus and resolved four hours post-LA administration, aligning with existing literature [7]. However, because the findings that can be observed in Horner’s syndrome can be precursors of high sympathetic blockade or cardiopulmonary arrest, vigilant follow-up is necessary from detection until the elimination of the block [8]. In most cases, it is transient and requires only conservative management along with reassurance. However, it may sometimes require imaging studies, such as MRI or CT scans, to rule out serious pathologies. Regarding the test dose, there is no specific regimen to test the epidural before using it for labor analgesia in our hospital. In this case, for example, 3 mL of the mixture in the bag (0.1% levobupivacaine + 2 mcg/mL fentanyl) was used to test the epidural before the patient began using it regularly. Others used 3 mL of 2% lidocaine followed by 6 mL of 0.25% levobupivacaine. However, we agreed that a test dose, per se, had no role in evidence-based practices, and every epidural dose could be considered a test dose [9]. Due to its benign self-limiting nature, many authors do not recommend avoiding using an epidural catheter if Horner’s syndrome occurs. Some others recommend performing a neurological evaluation of the patient with a history of this particularly rare complication before the placement of an epidural catheter to avoid subdural complications [10].

## Conclusions

This case highlights the rare but significant occurrence of Horner’s syndrome and unilateral upper limb paresthesia following labor epidural analgesia. It also underscores the need for further research into the mechanisms underlying the spread of LA in the epidural space during pregnancy. Diagnosing this condition is crucial to reassure patients about its transient and benign nature. It underscores the importance of recognizing the potential for high cephalad spread of LA in pregnant patients and the necessity for vigilant monitoring during epidural analgesia to promptly identify and manage such complications, which might include progression to total spinal and further respiratory or cardiac compromise. Also, further investigations like MRI and CT scans would be of great value to rule out possible anatomical anomalies in the epidural space.
